# Empagliflozin suppresses mitochondrial reactive oxygen species generation and mitigates the inducibility of atrial fibrillation in diabetic rats

**DOI:** 10.3389/fcvm.2023.1005408

**Published:** 2023-02-06

**Authors:** Takuya Koizumi, Masaya Watanabe, Takashi Yokota, Masumi Tsuda, Haruka Handa, Jiro Koya, Kotaro Nishino, Daishiro Tatsuta, Hiroyuki Natsui, Takahide Kadosaka, Taro Koya, Motoki Nakao, Hikaru Hagiwara, Rui Kamada, Taro Temma, Shinya Tanaka, Toshihisa Anzai

**Affiliations:** ^1^Department of Cardiovascular Medicine, Faculty of Medicine and Graduate School of Medicine, Hokkaido University, Sapporo, Japan; ^2^Institute of Health Science Innovation for Medical Care, Hokkaido University Hospital, Sapporo, Japan; ^3^Department of Cancer Pathology, Faculty of Medicine and Graduate School of Medicine, Hokkaido University, Sapporo, Japan; ^4^Department of Molecular Biology, Faculty of Medicine and Graduate School of Medicine, Hokkaido University, Sapporo, Japan; ^5^Kushiro City General Hospital, Kushiro, Japan; ^6^Hanaoka Seishu Memorial Hospital, Sapporo, Japan

**Keywords:** empagliflozin, diabetes, mitochondria, reactive oxygen species, SGLT2 inhibitor

## Abstract

**Introduction:**

Recent studies have demonstrated that sodium-glucose co-transporter-2 inhibitors (SGLT2-i) reduce the risk of atrial fibrillation (AF) in patients with diabetes mellitus (DM), in which oxidative stress due to increased reactive oxygen species (ROS) contributes to the pathogenesis of AF. We aimed to further investigate this, and examine whether the SGLT2-i empagliflozin suppresses mitochondrial-ROS generation and mitigates fibrosis.

**Methods:**

A high-fat diet and low-dose streptozotocin treatment were used to induce type-2 DM (T2DM) in Sprague-Dawley rats. The rats were randomly divided into three groups: control, DM, and DM treated with empagliflozin (30 mg/kg/day) for 8 weeks. The mitochondrial respiratory capacity and ROS generation in the atrial myocardium were measured using a high-resolution respirometer. Oxidative stress markers and protein expression related to mitochondrial biogenesis and dynamics as well as the mitochondrial morphology were examined in the atrial tissue. Additionally, mitochondrial function was examined in H9c2 cardiomyoblasts. Atrial tachyarrhythmia (ATA) inducibility, interatrial conduction time (IACT), and fibrosis were also measured.

**Results:**

Inducibility of ATA, fibrosis, and IACT were increased in rats with DM when compared to controls, all of which were restored by empagliflozin treatment. In addition, the rats with DM had increased mitochondrial-ROS with an impaired complex I-linked oxidative phosphorylation capacity. Importantly, empagliflozin seemed to ameliorate these impairments in mitochondrial function. Furthermore, empagliflozin reversed the decrease in phosphorylated AMPK expression and altered protein levels related to mitochondrial biogenesis and dynamics, and increased mitochondrial content. Empagliflozin also improved mitochondrial function in H9c2 cells cultured with high glucose medium.

**Discussion:**

These data suggest that empagliflozin has a cardioprotective effect, at least in part, by reducing mitochondrial ROS generation through AMPK signaling pathways in the atrium of diabetic rats. This suggests that empagliflozin might suppress the development of AF in T2DM.

## Introduction

Atrial fibrillation (AF) is the most common arrhythmia worldwide (1). The prevalence of AF is expected to increase by 2.5-times over the next 50 years (2). At the same time, diabetes mellitus (DM) has become one of the most common lifestyle disorders in world. DM and AF commonly coexist, and effective therapy to reduce the risk of AF in diabetic patients is needed; however, data regarding the benefit of glycemic control in new-onset AF remain scarce (3, 4).

Sodium-glucose co-transporter-2 inhibitors (SGLT2-i) are novel glucose-lowering drugs that act in a non-insulin-dependent manner (5). The EMPA-REG OUTCOME study revealed that empagliflozin significantly reduced the risk of major adverse cardiovascular (CV) events in patients with type-2 DM (T2DM) who were at high risk of CV events (6). The CANVAS Program, testing another SGLT2-i, canagliflozin, also demonstrated its effects on CV risk reduction (7). In addition, recent studies have suggested that SGLT2-i reduce the risk of AF in patients (8). Initial findings have suggested that SGLT2-i may prevent the electrical and structural remodeling of atria by ameliorating mitochondrial function (9), suppressing reactive oxygen species (ROS) generation, and inhibiting Na^+^/H^+^ exchanger activity (10, 11). However, the mechanisms pertaining to the beneficial effects of SGLT2-i in the prevention of AF have yet to be adequately investigated.

Oxidative stress refers to elevated intracellular levels of ROS, either due to excessive ROS generation or reduced ROS scavenging, which results in damage to lipids, proteins, and DNA (12). Oxidative stress and inflammation contribute to the pathogenesis of AF in patients with DM (4, 13). Indeed, previous studies have demonstrated that the reduction of ROS generation reduces AF inducibility in several experimental AF models (14, 15). Mitochondria are a major source of ROS as well as a source of intracellular energy production, and a recent study reported that empagliflozin improved atrial mitochondrial respiration in diabetic rats (9). However, rigorous evidence supporting the effects of SGLT2-i on mitochondrial oxidative stress in the atrial myocardium remains elusive. Here, we examined whether empagliflozin, an SGLT2-i, suppresses mitochondrial ROS generation and mitigates inflammatory as well as fibrotic remodeling of the atria, which could potentially result in reduced AF inducibility.

## Methods

### Ethical approval

This research protocol conformed to the Animal Care Guidelines for the Care and Use of Laboratory Animals of the Hokkaido University Graduate School of Medicine and was approved by the Animal Research Committee of Hokkaido University.

### Experimental animals

All experiments were performed using 8-week-old, male Sprague-Dawley rats. A high-fat diet (HFD) and low-dose streptozotocin (STZ) treatment were used to induce T2DM in rats. The HFD + low-dose STZ model, which demonstrates a progression from insulin resistance to hypoinsulinemia and hyperglycemia, mimics the natural pathogenesis of T2DM in humans. Therefore, it is suitable for investigating the pathogenesis of diabetic complications as well as testing the efficiency of anti-diabetic agents (9, 16). Typically, rats were fed with a HFD for 2–8 weeks in order to induce insulin resistance, and subsequently received a low-dose injection of STZ (20–40 mg/kg), resulting in increased blood glucose levels within 3–7 days after the STZ injection (16). In accord with the report by Shao et al. (9), we first tested a single dose of 30 mg/kg STZ to induce DM as a preliminary study; however, approximately half of rats died within a week possibly due to hyperglycemia-related complications. We thus reduced a dose of STZ to 25 mg/kg in accord with the report by Hou et al. (17).

Rats were randomly divided into three groups: control (*n* = 24), DM (*n* = 24), and DM + empagliflozin (30 mg/kg/day, *n* = 24). The empagliflozin dose was based on previous studies (9, 18). All rats were fed a normal chow diet for 1 week for acclimation. The DM and DM + empagliflozin groups were fed an HFD (60 kcal% fat, 20 kcal% carbohydrate, 20 kcal% protein; D12492; Research Diets, New Brunswick, NJ). The control group was fed a normal chow diet throughout the study. After 4 weeks of feeding, all animals were fasted overnight, and DM was induced by intraperitoneal injection of STZ (25 mg/kg) dissolved in citrate buffer at pH 4.5, into the DM and DM + empagliflozin groups. The control group was injected with the citrate buffer alone. One week following the STZ injection, the induction of DM was confirmed by blood glucose levels > 300 mg/dl. The same dose of STZ (25 mg/kg) injection as a second injection was repeated in rats whose blood glucose levels failed to meet the diagnostic criteria. In addition, if blood glucose levels failed to meet the diagnostic criteria even after second STZ injection, rats were excluded from our analyses. Blood glucose levels were measured using a glucometer (Glutest Every, Sanwa Kagaku Kenkyusho, Nagoya, Japan), and blood insulin levels were measured using LBIS Rat Insulin ELISA Kit (FUJIFILM Wako Pure Chemical Corporation, Osaka, Japan). The DM + empagliflozin group was treated with empagliflozin for 8 weeks. Empagliflozin was supplemented in HFD. Empagliflozin was supplied by Boehringer Ingelheim Pharma GmbH and Co. (Biberach, Germany). In the present study, we used different sets of rats (6–8 rats in each group) for each experiment and a total of 24 rats were used for whole experiment in each group, because it was impossible to conduct whole experiment in the same rat due to the limited samples.

### Experimental preparation

All animals were anesthetized with an intraperitoneal injection of a mixture (MMB) made from medetomidine hydrochloride (0.15 mg/kg, Kyoritsu Seiyaku, Tokyo, Japan), midazolam (2 mg/kg, Astellas Pharma, Tokyo, Japan), and butorphanol (2.5 mg/kg, Meiji Seika Pharma, Tokyo, Japan). Adequacy of anesthesia was monitored based on the disappearance of the pedal withdrawal reflex.

### Echocardiographic assessments

Following 8 weeks of treatment, transthoracic echocardiography was performed under anesthesia attained by means of an intraperitoneal injection of MMB. The rats were placed in the horizontal position, and echocardiographic parameters, including the left atrial (LA), dimension (LAD), interventricular septal thickness (IVST), left ventricular posterior wall thickness (LVPWT), left ventricular end-diastolic dimension (LVEDD), and left ventricular end-systolic dimension (LVESD), were obtained along the parasternal long-axis and short-axis views using an ultrasonographic system (APLIO 300 TUS-A300, TOSHIBA, Tokyo, Japan). Fractional shortening (FS) was calculated by measuring the percentage change in left ventricular diameter during systole.

### Langendorff-perfused heart

After confirming adequate anesthesia, heparin sodium (400 IU/kg) was intraperitoneally injected, and the hearts were quickly excised. The excised heart was mounted on a Langendorff apparatus and retrogradely perfused with Tyrode's solution (37°C) containing the following (in mmol/l): 143 NaCl, 5.4 KCl, 0.33 NaH_2_PO_4_, 5 HEPES, 5.5 glucose, 0.5 MgCl_2_, and 1.8 CaCl_2_ (pH 7.4 adjusted using NaOH) and gassed with 100% O_2_ until the heart rate was stable (19, 20).

### Electrophysiological study

We assessed the interatrial conduction time (IACT), effective refractory period (ERP), and atrial tachyarrhythmia (ATA) inducibility. An Ag/AgCl electrode was attached to the right atrium (RA) as a cathode to facilitate unipolar pacing, and a stainless steel microtube for perfusion was used as an indifferent anode. Two electrodes were attached to the LA appendage and left ventricle to record the electrogram. IACT was measured during RA pacing at cycle lengths of 150 and 200 ms. ERP was measured by introducing S2 extra-stimulus with 2-ms decrements following eight regulatory S1-S1 stimuli of 150 and 200 ms. It was defined as the longest S1-S2 interval at which S2 failed to induce a propagated response. We measured ERP twice in each heart, and the average was taken as the ERP used during statistical analysis. The induction of ATA was attained by burst pacing performed five times repeatedly, at a pacing cycle length ranging from 50 to 30 ms in 10-ms decrements for 3 s. ATA was defined as a rapid atrial response longer than 1s (20).

### Histology

Right atrial tissues were dissected from the hearts and stored in neutral buffered formalin for 24 h. The atrial tissue sections were stained with hematoxylin and eosin as well as Masson's trichrome stain to evaluate cardiomyocyte diameter and the extent of interstitial fibrosis, respectively. The cross-sectional area of cardiomyocytes was measured in the short-axis view. An average of 30 cardiomyocytes per animal were analyzed. In Masson's trichrome-stained sections, the area occupied by interstitial fibrosis was measured using BZ-X Analyzer software (KEYENCE, Osaka, Japan).

### Preparation of permeabilized fibers

After careful manual dissection of right atrial tissue, the fiber bundles were permeabilized by gentle agitation for 30 min in an ice-cold BIOPS solution (in mmol/l; 2.77 CaK_2_ EGTA, 7.23 K_2_ EGTA, 20 taurine, 6.56 MgCl_2_·6H_2_O, 5.77 Na_2_ ATP, 15 Na_2_ phosphocreatine, 20 imidazole, 0.5 dithiothreitol, and 50 MES hydrate; pH 7.1) with saponin (50 μg/ml), as described (21, 22). Following permeabilization, the fibers were rinsed twice by agitation for 10 min in an ice-cold respiration medium, MiR05 (in mmol/l; 110 D-sucrose, 60 K-lactobionate, 0.5 EGTA, 0.1% BSA, 3 MgCL_2_, 20 taurine, 10 KH_2_PO_4_, and 20 HEPES; pH 7.1).

### Mitochondrial respiratory capacity in the atrial muscle

We measured the mitochondrial respiratory capacity with non-fatty acid substrates in the permeabilized cardiac muscle fibers at 37°C using a high-resolution respirometer (Oxygraph-2k, Oroboros Instruments, Innsbruck, Austria), as described (21, 22). After the addition of the permeabilized atrial cardiac muscle fiber (2–3 mg) to the chamber filled with 2 ml of MiR05 in the Oxygraph-2k respirometer, the respiratory substrates and inhibitors were added in the following order: (1) glutamate (G; 10 mmol/l) and malate (M; 2 mmol/l) (complex I-linked substrates), (2) ADP (5 mmol/l), (3) succinate (S; 10 mmol/l) (complex II-linked substrates), (4) oligomycin (2 μg/ml) (a complex V inhibitor), (5) rotenone (0.5 μmol/l) (a complex I inhibitor), (6) antimycin A (2.5 mmol/l) (complex III inhibitor), (7) ascorbate (2 mmol/l) and N,N,N',N'-tert-methyl-p-phenyldiamine (TMPD; 0.5 mmol/l) (complex IV-linked substrates), and (8) sodium azide (10 mmol/l) (an inhibitor of cytochrome c oxidase). Complex IV capacity was calculated as the difference between the O_2_ consumption rates of ascorbate and TMPD with as well as without sodium azide to avoid the influence of auto-oxidation of TMPD. Respiratory rates were expressed as the O_2_ consumption rate normalized to the atrial muscle mass (pmol/s/mg wet weight of atrial muscle). The respiratory control ratio (RCR) was calculated as ADP-stimulated respiration (State 3 respiration)/non-ADP-stimulated respiration (State 2 or 4 respiration with oligomycin). Data acquisition and data analysis were performed using DatLab software (Oroboros Instruments).

### Mitochondrial ROS generation in the atrial muscle

We measured mitochondrial ROS generation along with mitochondrial respiratory capacity in the permeabilized atrial muscle fiber using a spectrofluorometer (Fluorescence LED2-Module, Oroboros Instruments) equipped with a respirometer, as described (23). Mitochondrial ROS generation was evaluated after the conversion of mitochondrial superoxide into hydrogen peroxide (H_2_O_2_) by the addition of superoxide dismutase (SOD). Before permeabilization of atrial muscle fibers, we added SOD (5 U/ml), horseradish peroxidase (1 U/ml), and Amplex UltraRed reagent (10 μmol/l, Thermo Fisher Scientific, Waltham, MA) to the chamber of the respirometer. H_2_O_2_ reacts with Amplex UltraRed in a 1:1 stoichiometry, catalyzed by horseradish peroxidase, which yields the fluorescent compound resorufin. The excitation and fluorescence wavelengths were 525 and 587 nm, respectively. The fluorescence of resorufin was continuously monitored along with measurements of mitochondrial respiratory capacity. The H_2_O_2_ generation rate was calibrated by the titration of H_2_O_2_ in 0.1 μmol/l increments before and after each addition of substrate, in order to eliminate the possible interference of substrates. The H_2_O_2_ generation rate was expressed as pmol/s/mg wet weight of atrial muscle.

### Oxidative stress in the atrial muscle

To assess oxidative stress in the atrial tissue, we measured the enzymatic activity of SOD using an SOD assay kit-WST (Dojindo, Kumamoto, Japan) and quantities of malondialdehyde (MDA), a lipid peroxidation product, using an MDA assay kit (Abcam, Cambridge, MA) as per the manufacturer's instructions. The right atrial tissue (5–6 mg) was used to measure activity of SOD and quantities of MDA, respectively.

### Mitochondrial enzymatic activities in the atrial muscle

A citrate synthase (CS) activity colorimetric assay kit (BioVision, Milpitas, CA) was used to biochemically assess the activity of CS, an enzyme involved in the tricarboxylic acid (TCA) cycle which occurs in the mitochondrial matrix. The right atrial tissue (5–6 mg) was used to measure the CS activity.

### Electron microscopy

Atrial tissues were fixed in 2.5% glutaraldehyde in 0.1 mmol/l phosphate buffer at 4°C. Tissues were then serially dehydrated in ethanol and embedded in epoxy resin. Consecutive ultrathin sections were mounted on copper grids and stained with 3% uranyl acetate and 0.2% lead citrate (24). They were then examined using a transmission electron microscope (H-7100, Hitachi, Tokyo, Japan). Mitochondria in the atrial cardiomyocyte were identified by their double membrane boundary and the presence of cristae, and their area was measured using ImageJ software.

### Quantitative real-time reverse transcription polymerase chain reaction

Gene expression levels were quantified by real-time RT-PCR as previously described (25). Ribonucleic acid (RNA) was extracted en masse from atrial tissue (8–10 mg) using QuickGene-810 (FujiFilm, Tokyo, Japan) as per the manufacturer's instructions. Complementary DNA (cDNA) was synthesized using a high-capacity cDNA reverse transcription kit (Applied Biosystems, Foster City, CA). A TaqMan quantitative PCR was performed using the StepOnePlus^TM^ Real-Time PCR System (Applied Biosystems, Waltham, MA) to amplify samples for transforming growth factor (TGF)-β, collagen type I, collagen type III, tumor necrosis factor (TNF)-α, interleukin (IL)-1β, and IL-6 cDNA. These transcripts were normalized using glyceraldehyde 3-phosphate dehydrogenase (GAPDH). The primers were purchased from Applied Biosystems.

### Western blotting and antibodies

Atrial tissues were harvested, snap frozen in liquid nitrogen, and stored at −80°C until use. For lysate preparation, atrial tissues (8–10 mg) were homogenized and dissolved in cell lysis buffer (Cell Signaling Technology, Danvers, MA) supplemented with Complete Protease Inhibitor Cocktail (Roche, Basel, Switzerland). Following centrifugation at 15,000 rpm for 20 min at 4°C, supernatants were separated into aliquots and stored at −80°C until the time of the assay. The protein concentrations were determined using a standardized colorimetric assay. Proteins were fractionated using SDS-PAGE, transferred to a polyvinylidene fluoride membrane, and blocked with 5% BSA or 5% milk for 1 h at room temperature (23–25°C) or overnight at 4°C. Target antigens were labeled overnight with primary antibodies at 4°C. Binding of the primary antibodies against nuclear factor (NF)-κB (#3034; dilution 1:1,000; Cell Signaling), phosphorylated NF-κB (#3037; dilution 1:1,000; Cell Signaling), AMP-activated protein kinase (AMPK) (#2532; dilution 1:1,000; Cell Signaling), pAMPK (#2535; dilution 1:1,000; Cell Signaling), peroxisome proliferator-activated receptor γ coactivator (PGC)-1α (#4259; dilution 1:1,000; Cell Signaling), mitochondrial transcription factor A (TFAM) (#sc-23588, dilution 1:1,000; Santa Cruz Biotechnology, Dallas, TX), mitofusin 1 (Mfn1) (#ab126575; dilution 1:1,000; Abcam, Cambridge, UK), mitofusin 2 (Mfn2) (#ab56889; dilution 1:1000; Abcam), optic atrophy 1 (OPA1) (#ab42364; dilution 1:1000; Abcam), dynamin-related protein 1 (Drp1) (#ab184247; dilution 1:1000; Abcam) was detected using specific horseradish peroxidase-conjugated secondary antibodies. Bands were detected using an enhanced chemiluminescence assay and quantified using ImageJ software (National Institutes of Health, Bethesda, MD). The band intensity for the protein being investigated was normalized to the intensity of GAPDH (Cell Signaling Technology) in each lane.

### H9c2 cell culture and measurement of cellular mitochondrial function

H9c2 cardiomyoblasts (CRL-1446^TM^, ATCC, Manassas, VA) were cultured in Dullbecco's Modified Eagle's Medium (25 mmol/l glucose, Sigma-Aldrich, St. Louis, MO) supplemented with 10% fetal bovine serum (Gibco) at 37°C with 5% CO_2_ in air (26). Then, H9c2 cells were maintained in the same medium (defined as high glucose [25 mmol/l]), or cultured in the same medium plus empagliflozin (10 μmol/l) (defined as high glucose + empagliflozin) or in the same medium but lowered glucose concentration (2.5 mmol/l) (defined as low glucose) for 24 h before measurement of cellular mitochondrial function (27). Approx. 30 min before the measurements, cells were trypsinized with 0.025% trypsin/PBS and suspended in MiR05 buffer, followed by cell count with Countess (Invitrogen). After the addition of cells (0.5–1.0 million cells) to the chamber (2 ml) of the Oxygraph-2k respirometer, we measured mitochondrial respiratory capacity and mitochondrial ROS generation with the same protocol except addition of digitonin (1.5 μmol/l) for cellular permeabilization before adding glutamate and malate, as described with minor modifications (28).

### Statistical analysis

Data are expressed as mean ± standard error (SE) or median (interquartile range) as appropriate. Statistical differences between the three independent groups were determined by one-way ANOVA or the Kruskal-Wallis test. For the *post-hoc* test, Tukey's or Dunn's multiple comparison test was performed. Differences were considered significant at *p* values < 0.05. Statistical analyses were performed using GraphPad Prism ver. 7 (GraphPad Software, San Diego, CA).

## Results

### Animal characteristics

Table 1 presents the characteristics of each group. Blood glucose levels in the DM group were significantly higher than those in the control group and were decreased by treatment with empagliflozin. There were no significant differences in body weights between the groups. The data of time course of changes in blood glucose levels and body weight are shown in Supplementary Figure 1. The number (%) of rats requiring the second STZ injection was comparable between DM and DM + EMPA groups [4/24 (16.7%) in DM vs. 4/24 (16.7%) in DM + EMPA, *p* = 1.00]. Supplementary Table 1 shows comparison of blood glucose levels, blood insulin levels and body weight between rats requiring single and double STZ injections. Compared to the controls, DM rats had a hypertrophied LV (characterized by increased IVST and LVPWT), whereas the increased LVPWT seemed to resolve to a significant extent following treatment with empagliflozin. There were no significant differences in LA or LV sizes and systolic function between the groups. Supplementary Table 2 shows both heart and right atrial weight.

**Table 1:** Characteristics of rats in three groups.

	**Control (*n =* 24)**	**DM (*n =* 24)**	**DM + EMPA (*n =* 24)**
Blood glucose, mg/dl	209 ± 8	374 ± 11^*^	201 ± 10^†^
Blood insulin, ng/ml	0.38 ± 0.02	0.25 ± 0.01	0.28 ± 0.01
Body weight, g	527 ± 15	515 ± 13	546 ± 12
LAD, mm	4.3 ± 0.1	4.3 ± 0.1	4.3 ± 0.1
IVST, mm	1.4 ± 0.1	1.5 ± 0.1^*^	1.4 ± 0.1
LVPWT, mm	1.4 ± 0.1	1.6 ± 0.1^*^	1.5 ± 0.1^†^
LVEDD, mm	7.9 ± 0.1	7.8 ± 0.2	8.0 ± 0.2
LVESD, mm	5.1 ± 0.1	4.8 ± 0.2	5.0 ± 0.1
FS, %	35.8 ± 0.9	38.4 ± 1.7	38.6 ± 0.9
Heart rate, bpm	278 ± 10	267 ± 10	274 ± 7

Values are expressed as mean ± standard error. ^*^p < 0.05: vs. Control, ^†^*p* < 0.05: vs. DM (One-way repeated ANOVA with *post hoc* Tukey's multiple-comparison test). LAD, left atrial dimension; IVST, interventricular septal thickness; LVPWT, left ventricular posterior wall thickness; LVEDD, left ventricular end-diastolic dimension; LVESD, left ventricular end-systolic dimension; FS, fractional shortening; DM, diabetes; EMPA, empagliflozin.

### Electrophysiology

**Figure 1:**
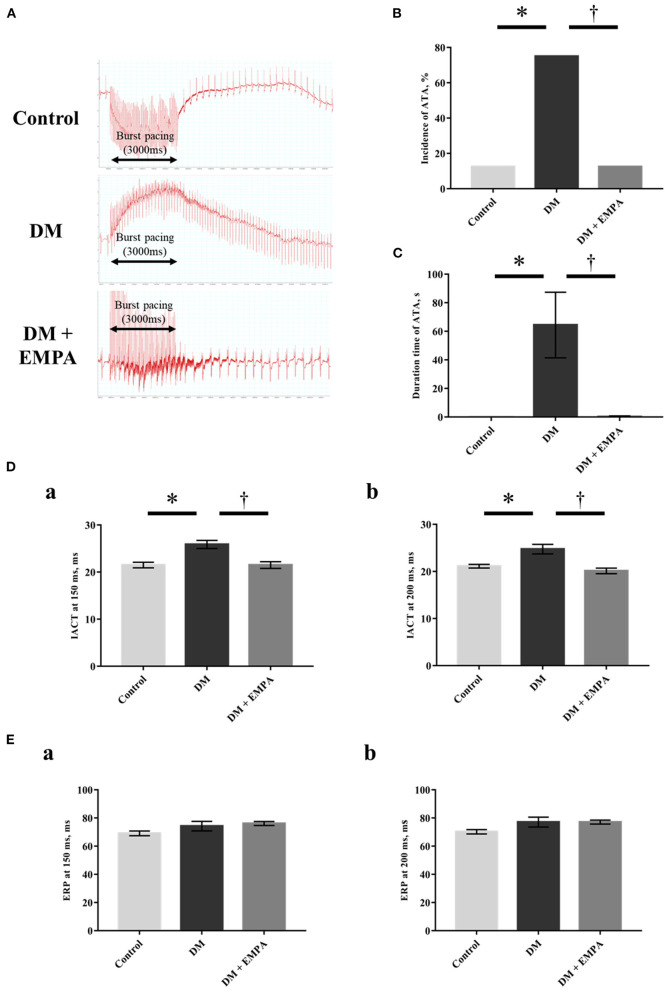
Electrophysiological study. **(A)** Representative electrogram traces obtained from rats in the three groups. **(B)** Incidence of atrial tachyarrhythmia (ATA). **(C)** Duration time of ATA. **(D)** Interatrial conduction time (IACT) at pacing cycle length of 150 ms (a) and 200 ms (b). **(E)** Effective refractory period (ERP) at pacing cycle length of 150 ms (a) and 200 ms (b). Data are expressed as mean ± SE (*n* = 8 per group). ^*^p < 0.05: vs. Control, ^†^*p* < 0.05: vs. DM (One-way repeated ANOVA with *post hoc* Tukey's multiple-comparison test and Fisher's exact test). DM, diabetes; EMPA, empagliflozin.

A representative electrocardiogram trace during the induction of ATA in each group is shown in Figure 1A. We observed both regular and irregular ATA in the induction experiment. We defined a regular ATA as atrial tachycardia (AT) and an irregular one as AF. In the control and the DM + empagliflozin group, ATA was induced in one of 8 rats (12.5%), which showed an irregular RR interval, defined as AF. In the DM group, ATA was induced in 6 of 8 rats (75%), of which 4 ATAs were defined as AF and the remaining 2 were as AT. ATA inducibility (Figure 1B), ATA duration (Figure 1C), and IACT (Figure 1D) were significantly increased in the DM group when compared to the control group, all of which was normalized following treatment with empagliflozin. There was no significant difference in the ERP (Figure 1E).

### Histological analysis of atrial muscle

**Figure 2:**
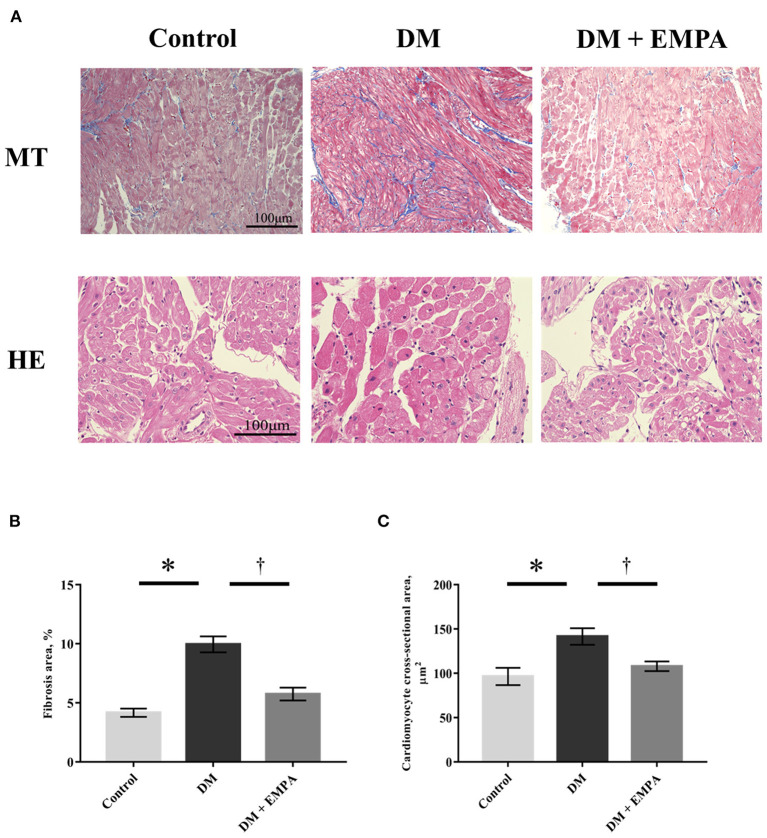
Histological study. **(A)** Representative images of right atrial tissue stained with Masson's trichrome (upper panels) and hematoxylin as well as eosin (bottom panels). **(B)** Area of fibrosis. **(C)** Cardiomyocyte cross-sectional area. Data are expressed as mean ± SE (*n* = 8 per group). ^*^p < 0.05: vs. Control, ^†^*p* < 0.05: vs. DM (One-way repeated ANOVA with *post hoc* Tukey's multiple-comparison test). MT, Masson's trichrome stain; HE, hematoxylin and eosin stain; DM, diabetes; EMPA, empagliflozin.

Masson's trichrome staining of the atrial muscle revealed that fibrotic area was more abundant in the DM group than in the control group, but the extent of fibrosis was lesser in the diabetic rats treated with empagliflozin (Figures 2A, B). Compared to the control group, the cardiomyocyte cross-sectional area was significantly larger in the DM group, which again seemed to be attenuated by empagliflozin treatment (Figure 2C).

### Fibrosis and inflammatory signaling in the atrial muscle

**Figure 3:**
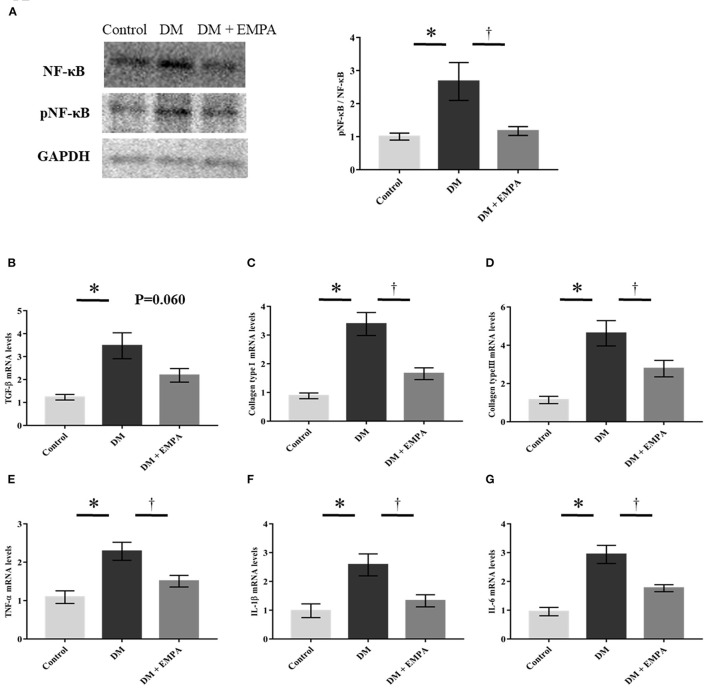
Studies on fibrotic and inflammatory signaling pathway. **(A)** Representative western blot results and analysis for the expression of NF-κB and phosphorylated NF-κB (pNF-κB). The value is represented as the ratio of pNF-κB to NF-κB (*n* = 6 per group). **(B–G)** Relative mRNA levels of TGF-β, collagen type I, collagen type III, TNF-α, IL-1β and IL-6 were measured by quantitative real-time RT-PCR (*n* = 8 per group). Data are expressed as mean ± SE. ^*^p < 0.05: vs. Control, ^†^*p* < 0.05: vs. DM (One-way repeated ANOVA *post hoc* Tukey's multiple-comparison test). NF-κB, nuclear factor-κB; GAPDH, glyceraldehyde 3-phosphate dehydrogenase; TGF-β, transforming growth factor-β; TNF-α, tumor necrosis factor-α; IL, interleukin; DM, diabetes; EMPA, empagliflozin.

As NF-κB plays a crucial role in the signaling pathways involved in fibrosis and inflammation, we investigated NF-κB signaling. As shown in Figure 3A, the phosphorylation of NF-κB, promoted by DM, was downregulated following treatment with empagliflozin. TaqMan quantitative PCR analysis revealed that mRNA expression of TGF-β, collagen type I, collagen type III, TNF-α, IL-1β, and IL-6 was markedly enhanced in the DM group when compared to the control group (Figures 3B–G), and treatment with empagliflozin significantly reduced the mRNA expression of collagen type I, collagen type III, TNF-α, IL-1β, and IL-6. TGF-β mRNA expression was comparable between the DM and DM + empagliflozin groups.

### Mitochondrial function in the atrial muscle

**Figure 4:**
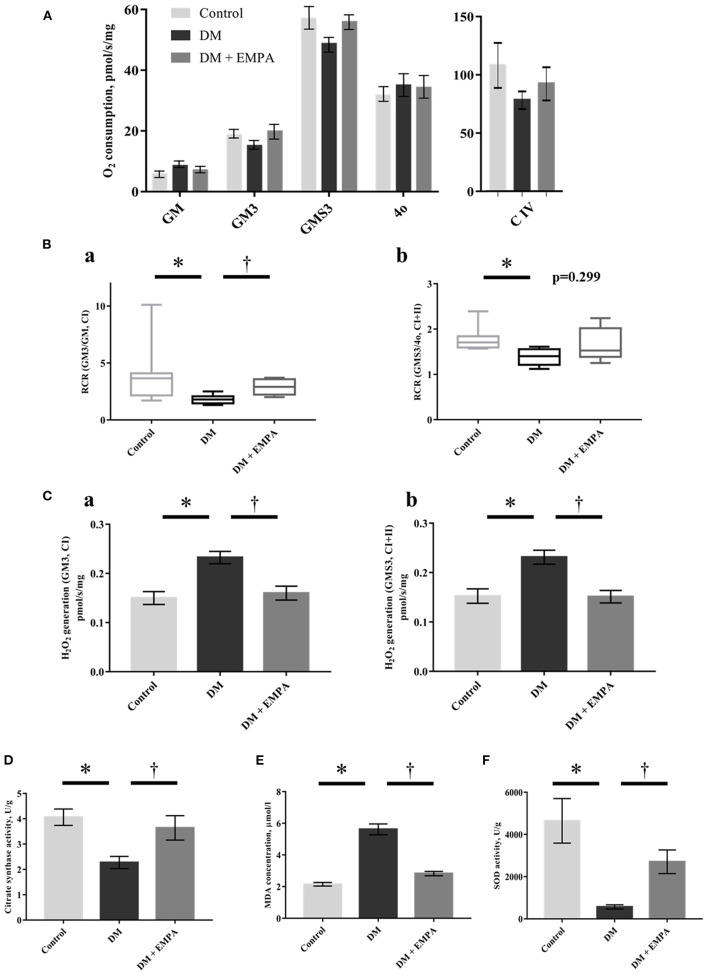
Effects of empagliflozin on mitochondrial respiratory capacity and ROS generation, as well as oxidative stress in the atrial tissue. **(A)** Summarized data of mitochondrial respiration during each state (*n* = 8 per group). **(B)** Respiratory control ratio (RCR) with CI-linked substrates (a) and CI+II-linked substrates (b). The line indicates median and error bars indicate the interquartile range (*n* = 8 per group). **(C)** Mitochondrial H_2_O_2_ generation with CI-linked substrates (a) and CI+II-linked substrates (b) (*n* = 8 per group). **(D)** Citrate synthase activity (*n* = 6 per group). **(E)** MDA concentration (*n* = 8 per group). **(F)** SOD activity (*n* = 6 per group). Data are expressed as mean ± SE or medians (interquartile range), as appropriate. ^*^p < 0.05: vs. Control, ^†^*p* < 0.05: vs. DM (One-way repeated ANOVA with *post hoc* Tukey's multiple-comparison test or Kruskal-Wallis test with *post hoc* Dunn's multiple comparison test). GM, state 2 respiration with glutamate + malate; GM3, state 3 respiration with glutamate and malate; GMS3, state 3 respiration with glutamate; malate and succinate; 4o, state 4 respiration with oligomycin; C, complex; H_2_O_2_, hydrogen peroxide; DM, diabetes; EMPA, empagliflozin.

The absolute values of mitochondrial respiration in each state are shown in Figure 4A. Diabetic rats had a lower mitochondrial RCR with complex I-linked or complex I + II-linked substrates than controls (Figure 4B). Empagliflozin treatment improved the complex I-linked RCR in the atrial muscle (Figure 4B). Mitochondrial H_2_O_2_ generation with either complex I-or complex I+II-linked substrates during state 3 (i.e., ADP-dependent state) was significantly elevated in the DM group when compared to the control group, which improved with treatment using empagliflozin (Figure 4C).

The enzymatic activity of CS was significantly reduced in the DM group when compared to that in the control group, and the administration of empagliflozin increased this enzymatic activity (Figure 4D).

### Oxidative stress in the atrial muscle

Consistent with the increased mitochondrial ROS generation, the atrial concentration of MDA was significantly higher in the DM group than in the control group, and empagliflozin treatment decreased MDA concentration to nearly the same level as that in control rats (Figure 4E). Furthermore, SOD activity was significantly decreased in the DM group when compared to that in the control group. This decrease was also reversed in the group that was administered empagliflozin (Figure 4F).

### Mitochondrial content in the atrial muscle

**Figure 5:**
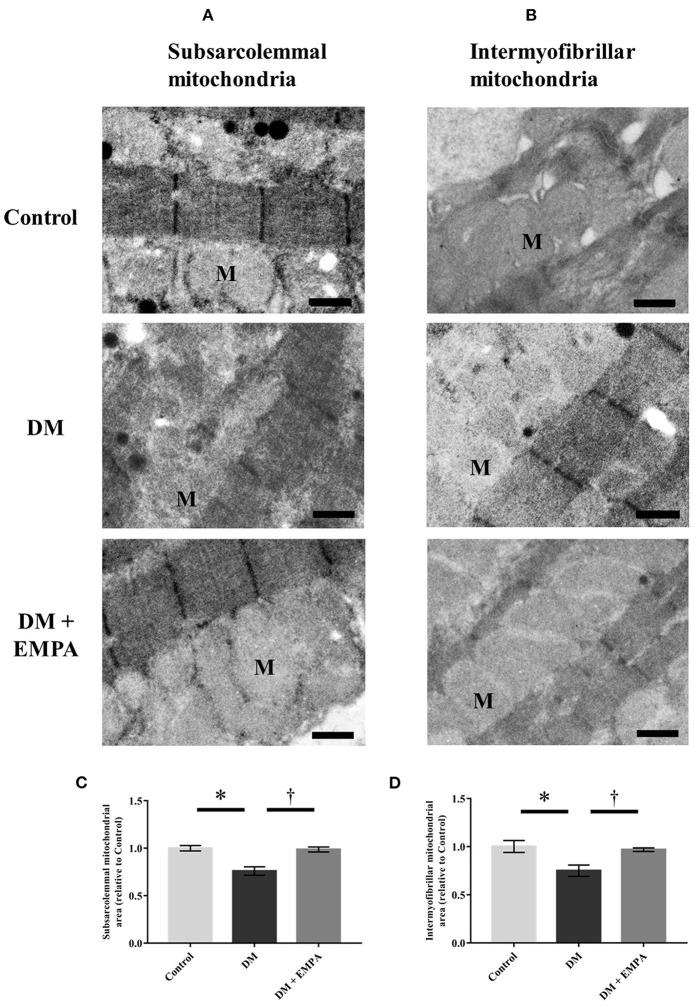
Morphological assessment of mitochondria in the atrial tissue. Representative electron microscopic images of subsarcolemmal [**(A)**; scale bar = 6 μm] and intermyofibrillar [**(B)**; scale bar = 6 μm] mitochondria. Summarized data of area of subsarcolemmal **(C)** and intermyofibrillar **(D)** mitochondria (*n* = 3 per group). Data are expressed as mean ± SE. ^*^p < 0.05: vs. Control, ^†^*p* < 0.05: vs. DM (One-way repeated ANOVA *post hoc* Tukey's multiple-comparison test). M, mitochondria; DM, diabetes; EMPA, empagliflozin.

To assess morphological changes of the mitochondria, we evaluated atrial mitochondria in rats using electron microscopy. Figures 5A, B show representative electron microscopic images of the subsarcolemmal and intermyofibrillar mitochondria. In the DM group, both subsarcolemmal and intermyofibrillar mitochondrial areas in the atrial tissue were significantly decreased compared to the control group (Figures 5C, D). Empagliflozin treatment ameliorated the reduced mitochondrial areas in the DM group (Figures 5C, D). However, there was no structural change in the atrial mitochondria of diabetic rats.

### Protein expression related to AMPK-PGC-1α signaling and mitochondrial dynamics in the atrial muscle

**Figure 6:**
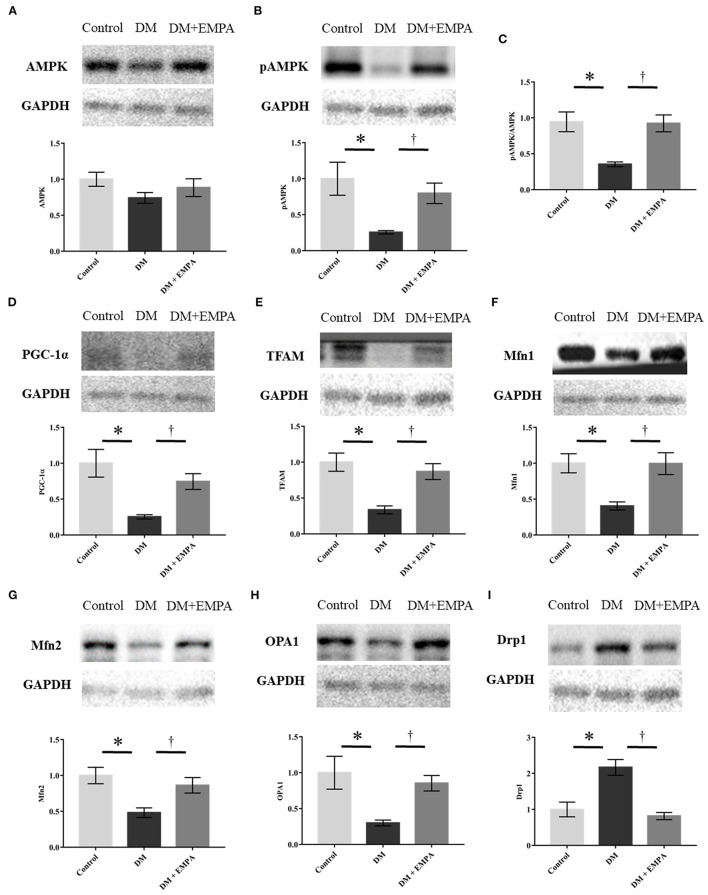
Western blotting analyses regarding mitochondrial biogenesis, fission, and fusion. **(A, B, D–I)** Representative western blot results and analyses for the protein expression of AMPK, phosphorylated AMPK, PGC1-α, TFAM, Mfn1, Mfn2, OPA1, and Drp1. **(C)** The value expressed is the ratio of pAMPK to AMPK. GAPDH was used as an endogenous control. Data are expressed as mean ± SE (*n* = 6 per group). ^*^p < 0.05: vs. Control, ^†^*p* < 0.05: vs. DM (One-way repeated ANOVA with *post hoc* Tukey's multiple-comparison test). pAMPK, phosphorylated AMP-activated protein kinase; PGC-1α, peroxisome proliferator-activated receptor γ coactivator-1α; Mfn, mitofusin; OPA1, optic atrophy 1; Drp1, dynamin-related protein 1; GAPDH, glyceraldehyde 3-phosphate dehydrogenase; DM, diabetes; EMPA, empagliflozin.

AMPK plays a major role in the signaling pathways related to energy metabolism and mitochondrial biogenesis. Although there was no significant difference in the protein expression of AMPK between the groups (Figure 6A), phosphorylated AMPK and its downstream molecules; PGC-1α and TFAM, were downregulated in the DM group, which improved upon treatment with empagliflozin (Figures 6B–E). Protein expression of Mfn1, Mfn2, and OPA1, all of which are related to mitochondrial fusion, was significantly downregulated, while Drp1, which is related to mitochondrial fission, was upregulated in the DM group when compared to the control group (Figures 6F–I). These changes in the atrial muscle of diabetic rats all seemed to be reversed to a large extent by the administration of empagliflozin (Figures 6F–I).

### Mitochondrial function in H9c2 cells

**Figure 7:**
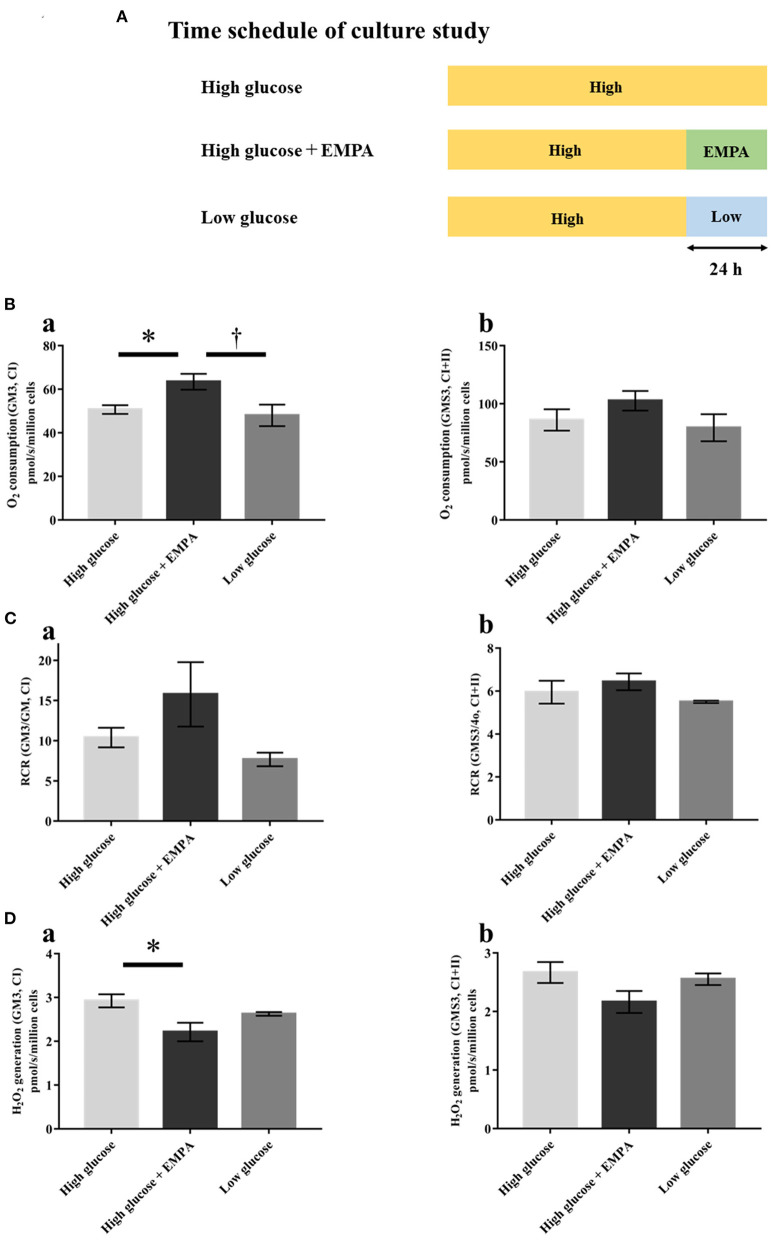
Effects of empagliflozin on mitochondrial respiratory capacity and ROS generation in H9c2 cells. **(A)** Time schedule of culture study [High glucose (25 mmol/l); *n* = 4, High glucose + EMPA (10 μmol/l); *n* = 3, Low glucose (2.5 mmol/l); *n* = 3]. **(B)** Mitochondrial H_2_O_2_ generation with CI-linked substrates (a) and CI+II-linked substrates (b). **(C)** O_2_ consumption with CI-linked substrates (a) and CI+II-linked substrates (b). **(D)** Respiratory control ratio (RCR) with CI-linked substrates (a) and CI+II-linked substrates (b). Data are expressed as mean ± SE. ^*^p < 0.05: vs. High glucose, ^†^*p* < 0.05: vs. High glucose + EMPA (One-way repeated ANOVA with *post hoc* Tukey's multiple-comparison test. Abbreviations are as defined in Figure 4.

We further measured the mitochondrial respiratory capacity and mitochondrial ROS generation in H9c2 cells to examine whether empagliflozin has a direct effect on cardiac mitochondria beyond glucose-lowering effect. Figure 7A shows the time schedule of the culture study. Empagliflozin treatment increased state 3 respiration with complex I-linked substrates (Figure 7Da) and reduced mitochondrial H_2_O_2_ generation with complex I-linked substrates during state 3 in H9c2 cells (Figure 7Ba). In contrast, there were no significant differences in mitochondrial respiratory capacity and mitochondrial H_2_O_2_ generation between the high glucose and the low glucose groups (Figures 7B–D), indicating that alteration in glucose concentration may not affect mitochondrial function in H9c2 cells.

## Discussion

In this study, we tested the cardioprotective ability of empagliflozin against atrial remodeling and explored the underlying mechanisms associated with mitochondrial function in a murine model of T2DM. The major findings are as follows: (1) empagliflozin seemed to reverse inducibility of ATA, and conduction slowing; (2) empagliflozin suppressed atrial remodeling which resulted from inflammatory and profibrotic signaling; (3) empagliflozin improved mitochondrial respiratory capacity, suppressed excessive generation of mitochondrial ROS, increased mitochondrial content, and reduced the enhanced oxidative stress in the atrial myocardium; (4) empagliflozin restored the altered atrial protein expression linked to mitochondrial biogenesis, mitochondrial fusion, and fission to levels similar to those in control rats.

### Atrial remodeling in T2DM

AF and T2DM are common diseases, and T2DM is an independent risk factor for the development of AF (3, 29). The pathogenesis of AF in T2DM includes structural, electrical, electromechanical, and autonomic remodeling (4, 30). Hyperglycemia induces enhanced oxidative stress in the atrium and other organs *via* different mechanisms (30). The key mediators of arrhythmogenic atrial remodeling in DM are oxidative stress and inflammation (30). Previous studies of atrial samples from diabetic patients have revealed excessive generation of ROS accompanied by impaired mitochondrial oxidative metabolisms (31). Oxidative stress activates NF-κB and enhances the expression of TGF-β1 and TNF-α, which can potentially result in atrial fibrosis. Much like previous reports, we also have observed that myocardial interstitial fibrosis is a hallmark of atrial structural remodeling, which leads to slowing down of electrical conduction, as well as electrical heterogeneity in the atrium, finally resulting in the induction and maintenance of AF (9, 20).

Our findings support the notion that advanced atrial fibrosis is a major contributor to the development of AF in patients with T2DM. In addition, we demonstrated that mitochondrial oxidative stress and pro-inflammatory signaling are enhanced in the diabetic atrium. Thus, a therapeutic strategy aimed at reducing mitochondrial ROS generation and inflammation in the atrium, may contribute to the prevention of AF in T2DM patients.

### The role of mitochondria in oxidative stress

Oxidative stress is defined as a state in which cell injury and the excessive generation of ROS *in vivo* overwhelms the cell's inherent antioxidant defenses, thus damaging proteins, lipids, and DNA. In cardiomyocytes, the mitochondria play a central role in energy metabolism and are major source of ROS, as well as NADPH oxidases, and NOS uncoupling (32–34). Indeed, a previous study demonstrated that mitochondrial dysfunction is linked to atrial remodeling, leading to postoperative AF in patients (35). Furthermore, recent studies have suggested that mitochondrial oxidative stress can promote AF *via* RyR dysfunction with Ca^2+^ leakage (from sarcoplasmic reticula) as well as inflammatory and profibrotic cytokine release (34, 36).

In diabetic hearts, oxidative stress and inflammation are implicated as the central mediators of AF (37–39). Glucose fluctuations, which are common in DM, promote ROS generation, and ROS-induced oxidation in the mitochondria can further exacerbate oxidative stress (40, 41). Hence, mitochondrial dysfunction and oxidative stress are closely related and a therapeutic approach targeting mitochondrial dysfunction holds potential as a novel treatment in AF.

### SGLT2-i may protect against atrial remodeling and the development of AF

SGLT2-i, originally developed to treat T2DM, have been shown to decrease CV death and hospitalization due to heart failure in these patients. Recent studies have also suggested that SGLT2-i reduces the risk of AF in patients with DM (8, 42). However, a previous randomized controlled study reported that intensive glycemic control did not affect the rate of new-onset AF (43). In addition, the cardioprotective benefits of SGLT2-i have also been demonstrated in non-diabetic patients (44, 45). These reports suggest that SGLT2-i improves the outcomes of patients independent of DM status and *via* mechanisms other than the lowering of blood glucose levels. The protective mechanism of SGLT2-i involves many aspects including both direct and indirect effects on the heart (46). In the present study, empagliflozin improved mitochondrial function in H9c2 cells, but alteration in glucose concentration did not change mitochondrial function. Given that there is no SGLT2 expression in the heart, empagliflozin may have direct effects on cardiac mitochondria independently of glucose levels.

Currently, several molecular and cellular mechanisms by which SGLT2-i protects the CV system have been identified (47–49). The reduction of oxidative stress is considered a potential mechanism for the suppression of cardiac remodeling by SGLT2-i, by balancing abnormal Na^+^ and Ca^2+^ levels and protecting mitochondrial function (48). A previous study by Li et al. reported that empagliflozin ameliorated myocardial oxidative stress by reducing NADPH activity in the ventricular myocardium of a diabetic mouse (50). Shao et al. reported that empagliflozin restored the reduced mitochondrial respiratory capacity and reversed atrial structural remodeling in HFD/STZ-induced DM rats (9); however, the effects of empagliflozin on mitochondrial oxidative stress in the atrial myocardium had not been examined before. In the present study, for the first time, we demonstrated that empagliflozin has the effect of reducing excessive generation of mitochondrial ROS and oxidative stress, leading to improved mitochondrial oxidative phosphorylation capacity in the atrial myocardium of diabetic rats.

### Underlying mechanisms of the effects of SGLT2-i on mitochondrial function

The master regulator of cellular energy homoeostasis, AMPK, is activated in response to stresses that deplete the cellular supply of ATP, such as low glucose, hypoxia, and ischemia. Importantly, AMPK tightly regulates endogenous cardioprotective signaling pathways (51, 52). The activation (i.e., phosphorylation) of AMPK mitigates the impaired expression of proteins involved in mitochondrial homeostasis, as well as antioxidant genes (53, 54), which influence the increase in mitochondrial biogenesis and the decrease in mitochondrial ROS generation (13). In addition, AMPK influences the homeostasis of mitochondrial dynamics through phosphorylation of the mitochondrial fission factor (13, 55).

As AMPK activation is impaired in diabetes, the AMPK signaling pathway is considered a potential candidate for the treatment of DM and more specifically, its cardiac complications (56). Moreover, previous studies have reported that altered AMPK activity is implicated in the pathogenesis of AF in diabetic as well as non-diabetic patients, and that AMPK activators mitigate cardiac remodeling, in addition to delaying the occurrence of AF (13).

In the current study, we revealed that empagliflozin enhanced AMPK phosphorylation that was likely already impaired, and its downstream dysregulation of PGC-1α and TFAM expression, leading to an improvement in mitochondrial biogenesis, respiratory function, as well as reduction in ROS generation. In addition, empagliflozin adequately counteracted the excessive upregulation of Drp1 and depletion of the mitochondrial fusion proteins Mfn1, Mfn2 and OPA1, as well as the morphological alteration of the mitochondria, suggesting that empagliflozin potentially influences mitochondrial dynamics *via* AMPK activation.

**Figure 8:**
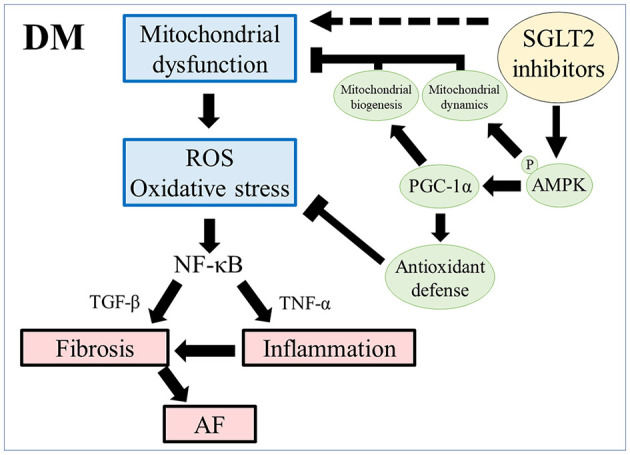
Scheme of atrial remodeling via mitochondrial dysfunction in rats with DM. ROS, reactive oxygen species; NF-κB, nuclear factor-κB; TGF-β, transforming growth factor-β; TNF-α, tumor necrosis factor-α; AF, atrial fibrillation; pAMPK, phosphorylated AMP-activated protein kinase; PGC-1α, peroxisome proliferator-activated receptor γ coactivator-1α.

Empagliflozin has been reported to reverse diabetic myocardial microvascular injury *via* AMPK-mediated inhibition of mitochondrial fission, and it ameliorated adverse cardiac remodeling and heart failure in a non-diabetic porcine model by enhancing myocardial energetics *via* AMPK activation (57, 58). Suggested mechanisms for AMPK phosphorylation include increasing the AMP/ATP ratio and the phosphorylation of liver kinase B1 (LKB1), an upstream activator of AMPK (57, 59). Our results are in line with those of previous studies and suggest that AMPK-mediated restoration of mitochondrial function plays a vital role in the mechanism of empagliflozin-induced inhibition of atrial remodeling. Taken together with our present findings and previous reports, we propose a possible mechanism for the cardioprotective effects of empagliflozin in the prevention of atrial remodeling in T2DM (Figure 8).

### Study limitations

Our study has certain limitations that need to be acknowledged. Firstly, the dose of empagliflozin used was greater than the clinical dose commonly used in humans, to ensure sufficient SGLT-2 inhibition. Secondly, because our study used murine models, our findings cannot be directly generalized in humans. Thirdly, we could not explore mechanisms other than mitochondrial biogenesis and dynamics, although recent experimental studies suggest that SGLT2-i can balance the abnormal homeostasis of Na^+^ and Ca^2+^ and/or mitigate the enhanced activity of calcium and calmodulin-dependent protein kinase II, which are expected to reduce mitochondrial Ca^2+^ levels (13). Fourthly, the following anesthetics; medetomidine hydrochloride (0.15 mg/kg), midazolam (2 mg/kg), and butorphanol (2.5 mg/kg) were used in all three groups of rats, as described (60). However, midazolam and butorphanol are likely to have inhibitory effects on mitochondrial complex I, II, III, and apoptosis (61, 62). Although these anesthetics were equally administered to each rat with same doses in all groups, we could not completely exclude the possibility of inhibitory effect of anesthetics on atrial mitochondrial function in rats. Finally, we did not comprehensively investigate cardioprotective mechanism of empagliflozin in diabetes, further mechanistic studies are needed to assess beneficial effects of empagliflozin on atrial mitochondria.

## Conclusion

Empagliflozin treatment reduced mitochondrial oxidative stress and prevented atrial remodeling in a murine model of T2DM. Empagliflozin restored impaired mitochondrial biogenesis and dynamics, most likely *via* AMPK-mediated pathways. Our findings highlight the therapeutic potential of empagliflozin in prevention of AF in patients with T2DM.

## Data availability statement

The original contributions presented in the study are included in the article/Supplementary material, further inquiries can be directed to the corresponding author.

## Ethics statement

This research protocol conformed to the Animal Care Guidelines for the Care and Use of Laboratory Animals of the Hokkaido University Graduate School of Medicine and was approved by the Animal Research Committee of Hokkaido University.

## Author contributions

TKoi and MW conceived the idea of the study and drafted the original manuscript. TKoi and TY developed the statistical analysis plan and conducted statistical analyzes. TKoi, MW, and TY contributed to the interpretation of the results. All authors reviewed the manuscript draft, revised it critically on intellectual content, and approved the final version of the manuscript to be published.
