# Supplementary figures and images for: Empagliflozin suppresses mitochondrial reactive oxygen species generation and mitigates the inducibility of atrial fibrillation in diabetic rats
Source: Front Cardiovasc Med. 2023 Feb 6;10:1005408. doi: 10.3389/fcvm.2023.1005408 (PMC9940756; doi:10.3389/fcvm.2023.1005408)

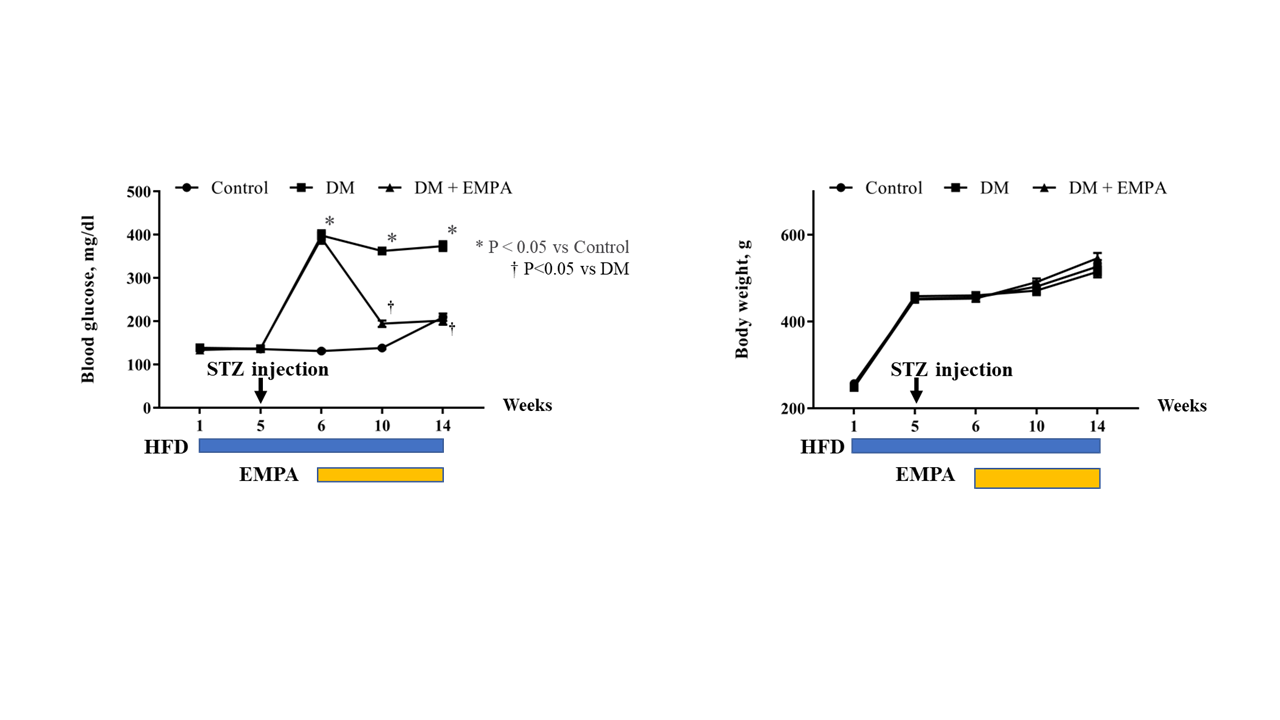

Supplement: Supplementary file 1 [file Image_1.TIF]
