# Supplementary material for: Empagliflozin suppresses mitochondrial reactive oxygen species generation and mitigates the inducibility of atrial fibrillation in diabetic rats
Source: Front Cardiovasc Med. 2023 Feb 6;10:1005408. doi: 10.3389/fcvm.2023.1005408 (PMC9940756; doi:10.3389/fcvm.2023.1005408)
Supplement: Supplementary file 2 [file Table_1.DOCX]

**Supplemental material**

***Original research article***

**Empagliflozin Suppresses Mitochondrial Reactive Oxygen Species Generation and Mitigates the Inducibility of Atrial Fibrillation in Diabetic Rats**

**Supplemental Figure legend**

**Supplemental Fig. 1** Time course of changes in blood glucose and body weight. DM, diabetes mellitus; EMPA, empagliflozin; HFD, high fat diet; STZ, streptozotocin.

**Supplemental Table 1** Comparison of blood glucose levels, blood insulin levels and body weight between rats requiring single and double STZ injections

| **DM** | | | |
| --- | --- | --- | --- |
|  | Single STZ injection, n = 20 | Double STZ injections, n = 4 | p |
| Blood glucose, mg/dl | 371 ± 11 | 384 ± 43 | 0.398 |
| Blood insulin, ng/ml | 0.25 ± 0.01 | 0.25 ± 0.01 | 0.473 |
| Body weight, g | 514 ± 15 | 517 ± 33 | 0.478 |
| **DM + EMPA** | | | |
|  | Single STZ injection, n = 20 | Double STZ injections, n = 4 | p |
| Blood glucose, mg/dl | 203 ± 10 | 193 ± 26 | 0.359 |
| Blood insulin, ng/ml | 0.28 ± 0.01 | 0.28 ± 0.01 | 0.488 |
| Body weight, g | 541 ± 14 | 573 ± 23 | 0.133 |

Values are expressed as mean ± standard error. DM, diabetes mellitus; EMPA, empagliflozin; STZ, streptozotocin.

**Supplemental Table 2** Heart and right atrial weight in three groups

|  | Control  n = 8 | DM  n = 8 | DM + EMPA  n = 8 | p |
| --- | --- | --- | --- | --- |
| Heart weight, mg | 1342 ± 27 | 1402 ± 34 | 1415 ± 30 | 0.229 |
| Right atrial weight, mg | 44.3 ± 1.0 | 48.3 ± 1.2 | 45.5 ± 1.0 | 0.874 |

Values are expressed as mean ± standard error. DM, diabetes mellitus; EMPA, empagliflozin.
